# Multicenter questionnaire survey for sporadic inclusion body myositis in Japan

**DOI:** 10.1186/s13023-016-0524-x

**Published:** 2016-11-08

**Authors:** Naoki Suzuki, Madoka Mori-Yoshimura, Satoshi Yamashita, Satoshi Nakano, Ken-ya Murata, Yukie Inamori, Naoko Matsui, En Kimura, Hirofumi Kusaka, Tomoyoshi Kondo, Itsuro Higuchi, Ryuji Kaji, Maki Tateyama, Rumiko Izumi, Hiroya Ono, Masaaki Kato, Hitoshi Warita, Toshiaki Takahashi, Ichizo Nishino, Masashi Aoki

**Affiliations:** 1 Department of Neurology, Tohoku University Graduate School of Medicine, 1-1 Seiryo-machi, Aoba-ku, Sendai, 980-8574 Japan; 2 Department of Neurology, National Center Hospital, National Center of Neurology and Psychiatry (NCNP), Tokyo, Japan; 3 Department of Neurology, Graduate School of Medical Sciences, Kumamoto University, 1-1-1 Honjo, Chuo-ku, Kumamoto Japan; 4 Department of Neurology, Osaka City General Hospital, 2-13-22 Miyakojima hondoori, Miyakojima-ku, Osaka Japan; 5 Department of Neurology, Wakayama Medical University, Wakayama, Japan; 6 Department of Neurology and Geriatrics, Kagoshima University Graduate School of Medical and Dental Sciences, Kagoshima, Japan; 7 Department of Clinical Neuroscience, Institute of Biomedical Sciences, Tokushima University Graduate School, Tokushima, Japan; 8 Department of Neurology, Kansai Medical University, 2-5-1, Shin-machi, Hirakata, Osaka Japan; 9 Department of Neurology, National Hospital Organization Sendai-Nishitaga National Hospital, Sendai, Japan; 10 Department of Neuromuscular Research, National Institute of Neuroscience, Tokyo, Japan; 11 Department of Genome Medicine Development, Medical Genome Center, National Center of Neurology and Psychiatry (NCNP), Tokyo, Japan

**Keywords:** Sporadic inclusion body myositis, Multicenter survey, Questionnaire, Aging, Muscle disease

## Abstract

**Background:**

Sporadic inclusion body myositis (sIBM) is the most prevalent acquired muscle disease in the elderly. sIBM is an intractable and progressive disease of unknown cause and without effective treatment. The etiology of sIBM is still unknown; however, genetic factors, aging, lifestyles, and environmental factors may be involved. The purpose of this study is to elucidate the cross-sectional profile of patients affected by sIBM in Japan.

**Methods:**

We surveyed patient data for 146 cases diagnosed at a number of centers across Japan. We also issued a questionnaire for 67 patients and direct caregivers to further elucidate the natural history of the disease.

**Results:**

The mean age at the onset was 63.4 ± 9.2 years. The mean length of time from the onset to diagnosis was 55.52 ± 49.72 months, suggesting that there is a difficulty in diagnosing this disease with long-term consequences because of late treatment. 73 % described the psychological/mental aspect of the disease. The most popular primary caregiver was the patient’s spouse and 57 % patients mentioned that they were having problems managing the finances.

**Conclusions:**

Through these surveys, we described the cross-sectional profiles of sIBM in Japan. Many patients described psychological/mental and financial anxiety because of the aged profile of sIBM patients. The profiles of sIBM patients are similar to those in Western countries.

## Background

Sporadic inclusion body myositis (sIBM) is the most common form of inflammatory myopathy in those over the age of 50 years in Western countries [1–3]. Muscle weakness and atrophy in the quadriceps, wrist flexor, and finger flexors are typical neurological findings of sIBM. Muscle biopsy typically reveals endomysial inflammation, invasion of mononuclear cells into non-necrotic fibers, and rimmed vacuoles, suggesting that inflammation and degeneration are co-existed in the pathological mechanism. The prevalence in Caucasians ranges between 4.9 and 14.9 per million but is only 1.07 in Turkey [4]. We previously performed a retrospective survey of Japanese patients diagnosed with sIBM at the National Center of Neurology and Psychiatry (NCNP) [5]. Even though the awareness of sIBM by physician after 1970s contributes to the detection bias, the increasing incidence of sIBM in Japan was followed by the rapid change in dietary habits from a traditional style to a Westernized diet after World War II, fascinating the speculation that this change in dietary habits might have an influence on the increasing number of patients with sIBM in Japan. Other groups have also reported that the number of Japanese patients with sIBM appears to have increased in recent years [6]. It is important to describe the detail of the clinical phenotypes in Japan to elucidate the influence of dietary components and genetic factors on the pathophysiology of sIBM. To further understand the cross-sectional profile of patients with sIBM in Japan, we surveyed patient data for 146 cases gathered through a multicenter study in Japan for the first time. We also performed a questionnaire survey of 67 patients and their direct caregivers to elucidate the natural history of the disease and aspects relating to anxiety. Through these surveys, we described the cross-sectional profiles of sIBM in Japan.

## Methods

### Participants and procedure

#### Multicenter survey

**Fig. 1:**
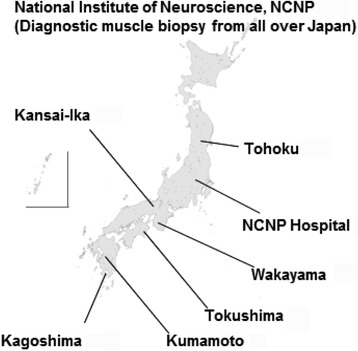
Patients’ distribution and collaborating institutes for Multicenter survey. A total of 146 patients were included from 8 institutes

This was a cross-sectional study targeting patients with sIBM registered at the collaborating institutes, which received patients with myopathy in Japan. The detailed information was gleaned from 146 patients diagnosed between January 2000 and December 2009 at 8 institutes, which were involved in the multicenter survey (Fig. 1). Only patients with “definite” or “probable” sIBM by clinical and biopsy criteria [3] were included in the analysis. Biopsies were re-evaluated, and the pathological diagnosis of sIBM was confirmed. All patient data including clinical and pathological information were registered by doctors. Each of the attending neurologists completed information pertaining to past medical history, family history, and data from medical records (biopsy findings, laboratory, and physiological data).

#### Questionnaire for patients and caregivers

We sent a postcard survey to neurologists in Japan asking whether the sIBM patients they are following could cooperate with the questionnaire survey. We distributed a registered self-administered questionnaire to the 67 patients, who agreed to reply through their attending physician between October 2010 and January 2011, and provided an explanation of the purpose of the study. It was emphasized that participation in the study was completely voluntary. Patients returned the questionnaires in sealed envelopes by themselves to ensure confidentiality. The protocol for this study was approved by the Ethics Committee of Tohoku University School of Medicine. Items in the questionnaire included the past medical history, complications, family medical history, disease onset, ambulation status, and results of muscle biopsy. The questionnaire included informations on name, age, height, weight, and lifestyle. The structure of the questionnaires for the patients and caregivers are listed in Table 1.

**Table 1:** Lists of questions for the patients and caregivers for questionnaire study

Basic information	Hospital
	Date
	Doctor’s name
	Name
	Date of birth and age
	Sex
	Address/Phone/E-mail
Life/Past History	Development
	Exercise Capacity at School
	Works
	Preference
Symptoms	Initial symptom
	Milestones: wheelchair, cane
	Mental/psychological stress
	Economic matters
Diagnosis	Age at admission
	Method of diagnosis
	Family history
For Caregiver	Activities of daily life
	Mental/psychological stress

### Data analysis

Data were summarized using descriptive statistics, including mean, standard deviation (SD), median, range, frequency, and percentage.

## Results

### Multicenter analysis

To elucidate the cross-sectional profile of patients with sIBM in Japan, we surveyed patient data for 146 cases in Japan. The demographic profile of the patients is described in Table 2.

**Table 2:** Patients characteristics for Multicenter survey

		Number	Percent	Mean ± SD	Minimum	Median	Maximum
Sex	Male	83	57				
	Female	63	43				
Age at onset (years)		137	94	63.43 ± 9.18	40	65	81
Duration from onset to diagnosis (months)		143	98	55.52 ± 49.72	3	36	288
CK (IU/L)		141	97	532.85 ± 371.33	30	453	2401

The normal range for CK is 62–287 IU/L for males and 45–163 IU/L for females

**Fig. 2:**
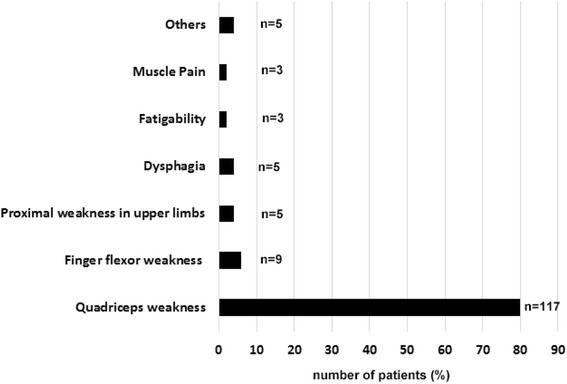
Initial symptoms of this cohort for Multicenter survey. The most common initial symptom was quadriceps weakness, including difficulty in climbing stairs and standing up from the chair. Some patients had weakness of finger flexor muscles and dysphagia. A small number of patients complained about muscle pain

In our cohort, the prevalence of male was higher than that of females (male: *n* = 83, female *n* = 63) (Table 2). The mean age at the onset was 63.43 ± 9.18 (range, 40–81) years. Dysphagia was observed in 29 patients (23.2 %). The involvement of flexor finger muscles was observed in 17 patients at the time of the survey. The most common initial symptom was weakness of the proximal lower muscles, including quadriceps femoris (117 patients, 80 %) (Fig. 2). Weakness of finger flexors (*n* = 9), shoulder girdle muscle (*n* = 5), muscle pain (*n* = 3), general fatigue (*n* = 3), and dysphagia (*n* = 5) were also noted. Apparent laterality of the muscle weakness was described in 16 cases. Family history of myopathy including familial inclusion body myopathy [7] was observed in 5 cases. Cognitive impairment was not observed in these 146 cases.

**Fig. 3:**
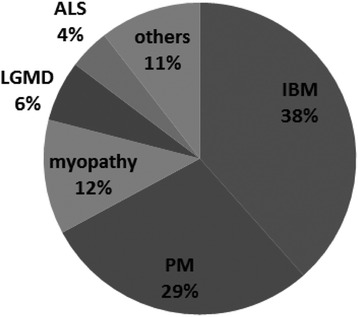
Presumptive diagnosis before muscle biopsy for the Multicenter survey. The clinical diagnosis of sIBM was confirmed in only 38 % patients. The others were PM (29 %), unclassified myopathy (12 %), LGMD (6 %), and ALS (4 %)

The clinical diagnosis before muscle biopsy included PM (*n* = 41), unclassified myopathy (*n* = 17), ALS (*n* = 6), and LGMD (*n* = 9). The presumptive diagnosis of sIBM was found in only 55 patients (Fig. 3).

In the laboratory data, serum creatine kinase level was 532.85 ± 371.33 (range, 30–2401). Antibodies against hepatitis B virus (HBV) (*n* = 3), HCV (*n* = 16), and HTLV-1 (*n* = 8) were observed. In muscle biopsy, necrotic fibers were found in 88 cases. COX negative fibers were found in 63 cases, although there are some COX negative fibers in aged muscles. Cellular infiltration in non-necrotic muscle fibers was observed in 67 cases. An increased amount of adipose tissue was observed in 40 cases. Cellular infiltration in the endomysium was present in 117 cases.

The mean length from disease onset to the time of diagnosis was 55.52 ± 49.72 (range, 3–288) months, suggesting that it is difficult to promptly diagnose this disease because of its clinical course.

**Fig. 4:**
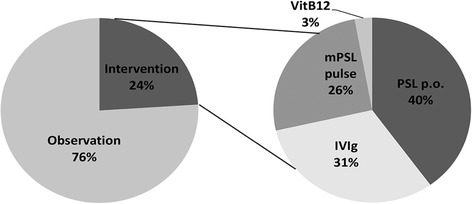
Interventions for this cohort for Multicenter survey. In total, 24 % patients underwent some type of intervention, whereas 76 % patients were allowed to follow the natural course of disease. Among those who received some form of intervention, prednisolone (PSL; 40 %), intravenous immunoglobulin (IVIg; 31 %), intravenous methyl-prednisolone (mPSL; 26 %), and vitamin B12 (VitB12; 3 %) were executed with limited effect

In total, 24 % patients underwent an intervention of some kind, whereas 76 % patients followed the natural course of the disease. Among those who underwent some type of intervention, prednisolone (40 %), intravenous immunoglobulin (IVIg; 31 %), intravenous methylprednisolone (26 %), and vitamin B12 (VitB12; 3 %) were administered (Fig. 4). Depending on the subjective judgment of the attending physician, 57 % showed no change and 7 % showed worsening of the disease among patients with treatment. Thirty-six percent of patients were reported to show improvement to some extent, even if only temporarily. The effect of these treatments should be reexamined in the organized study design.

### Questionnaire for patients and caregivers

To elucidate the natural history of the disease and to measure emotions in both patients and caregivers, we issued a questionnaire for both patients and caregivers of these patients. The profile of the patients is described in Table 3.

**Table 3:** Patients characteristics for questionnaire study

		Number	Percent	Mean ± SD	Minimum	Median	Maximum
Age at the survey (years)		67	100	73.20 ± 7.15	53	73	90
Sex	Male	49	73				
	Female	18	27				
Age at onset (years)		67	73	64.47 ± 8.21	40	65	80

**Fig. 5:**
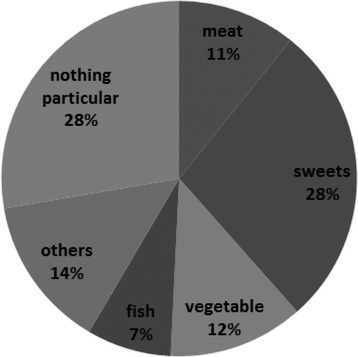
Preference of food in the questionnaire study. There was a wide preference of food, including meat, sweets, vegetables, and fish

The mean age at examination was 73.20 ± 7.15 years. The age at onset as described by the patients was 64.47 ± 8.21 years. The development and growth of cases were unremarkable. There was a wide variety in food preference, including meat, sweets, vegetables, and fish (Fig. 5). The percentage of patients who smoked was reported to be 54.5 %. The initial symptoms are similar to the survey findings obtained by the neurologist. The duration of milestone symptoms after the onset is described in Table 4. The inability to stand independently occurred after an average of 4.6 years. The average time of being wheelchair-bound was 7.3 years from the onset and that of being electrical wheelchair-bound was 13.7 years. The average time from onset to the inability to open a plastic bottle was 6.6 years. Furthermore, the time from onset until the patients were unable to wash their own face was 7.2 years.

**Table 4:** Milestones from questionnaire study

	Unable to stand up without assistance	Wheelchair bound	Electric wheel chair	Unable to open bottle	Unable to wash face
Number of patients	53	23	6	43	12
Average duration (years)	4.6	7.3	13.7	6.6	7.2
SD	4.4	4.9	7.3	5.9	4.3

**Fig. 6:**
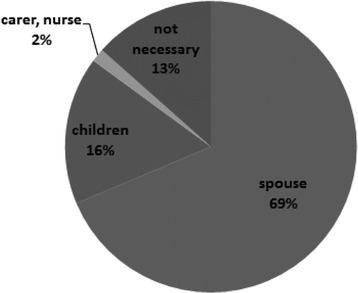
Patient caregiver results from the questionnaire study. The most popular primary caregiver was the spouse of the patient (69 %)

For questions relating to anxiety, 73 % described to the psychological/mental aspect of the disease. The most popular primary caregiver was the patient’s spouse (Fig. 6). As sIBM tends to occur from mid-to-older age, the partner of the patient often had no physical strength and may have had disease. Some patients complained about the bleak outlook of the sIBM therapy. With reference to the financial aspects of the disease and how it affected the household, 57 % patients mentioned that they were having problems managing the finances.

## Discussion

We surveyed patient data for 146 cases diagnosed at a number of centers across Japan to elucidate the cross-sectional profile of patients affected by sIBM in Japan. We also issued a questionnaire for 67 patients and direct caregivers to further elucidate the natural history of the disease. Through these surveys, we described the cross-sectional profiles of sIBM in Japan for the first time in the Asian countries. We found the same issues in the clinical practices of sIBM in Japan similar to Western countries.

Interestingly, a rather high percentage of patients were seropositive for HCV (*n* = 16) and HTLV-1 (*n* = 8) in our multicenter survey, corroborating the interaction between viral infection and the pathophysiology of sIBM, as mentioned previously. Eleven patients with HTLV-1–associated IBM were reported in the endemic area in Japan [8]. The prevalence of HTLV-1 infection in sIBM is higher than in the general population, compared with the prevalence in general in Japanese people aged 60 to 64 years in 2006–2007, which was estimated to be 1.5 % in men and 1.7 % in women [9]. Moreover, Uruha et al. reported that a significantly higher number of patients with sIBM (28 %) had anti-HCV antibodies than patients with polymyositis (4.5 %; odds ratio, 8.2) and the general Japanese population in their 60s (3.4 %) [9]. A pathomechanistic link between sIBM and HTLV1/HCV infection merits further research.

For the purpose of early intervention in clinical trials, early diagnosis of disease is important. Some of the patients in our study visited several physicians and the average time from date of onset to diagnosis was 55.52 months. Some patients show isolated weakness of finger flexors 3 years after onset but were diagnosed with sIBM in muscle biopsy 6 years after onset [10]. Some patients underwent repeat biopsy of the muscle because of the lack of findings related to rimmed vacuoles or other pathological markers. It is important to evaluate which muscle should be biopsied using CT and MRI. Currently, the utility of a new auto-antibody targeting cytosolic 5′-nucleotidase 1A (cN-1A) in the serum of patients with sIBM has recently been published [11]. The diagnostic utility for differentiating between sIBM, other forms of myositis, and other neuromuscular diseases requires further examination. Reliable positive markers for sIBM are necessary for the early diagnosis in future clinical trials.

As shown in Fig. 5, clinical diagnosis tends to be different from sIBM in early diagnosis. The importance of muscle biopsy is integral to the correct diagnosis. A clinical trial of bimagrumab is ongoing [12]. Transforming growth factor beta superfamily signaling, at least through ActRII, is implicated in the pathophysiology of sIBM. The inhibition of ActRII increased muscle mass and function in this pilot trial, thus, offering a potential novel treatment of sIBM [13]. Benveniste et al. reported that 71 (52 %) patients received immunosuppressive treatments such as prednisolone in 91.5, and 64.8 % were treated with other immunomodulatory drugs, including IVIgs, methotrexate, or azathioprine, for a median duration of 40.8 months [14]. This study confirms immunosuppressive treatments do not ameliorate the natural course of disease, thus, confirming findings from smaller studies. The decision to terminate conventional immunosuppressive treatment might be considered if there is no apparent beneficial effect after the trial, during a certain period of therapy.

In one of the abovementioned studies [15], euthanasia was requested by three patients, and in another three, continuous deep sedation was applied. The fact that end-of-life care interventions were used in six patients (13 %) reflects the severe disability and loss of quality of life at the end stage of this disease [15]. sIBM is a chronic progressive disorder, leading to major disabilities at the end stage of the disease because of extensive muscle weakness. Our questionnaire survey also revealed several qualitative aspects relating to caregivers who were typically spouses, and the difficulty in managing the disease because of its long duration (Fig. 6). Clearly, this impacts upon caregivers who themselves require societal support.

It is notable that 73 % of survey respondents described the anxiety and psychological/mental aspect of the disease. Developed countries, including Japan, have an aged society, and at mid-to-older age the partners of sIBM patients often lack physical strength and may have a disease. Some patients complained about the bleak outlook of sIBM therapy. A total of 57 % of patients mentioned problems managing finances. In Japan, there was no financial support for patients affected by sIBM at the time of the questionnaire; support for patients with intractable disease started in 2015. It is highly important to prepare social resources in developed countries for rare intractable diseases, including sIBM.

The present study has several limitations. First, the study used a retrospective and cross-sectional design, which cannot determine causal relationships. A longitudinal study should be conducted to address this issue. One group performed a follow-up study of 64 patients with sIBM who participated in a national epidemiological study in the Netherlands [15]. Case histories were recorded and manual and quantitative muscle tests as well as laboratory tests were performed at baseline and 12 years (median) after the first outpatient visit. Forty-six patients died during the follow-up period. The 15 surviving patients had a mean disease duration of 20 years. The mean decline in strength was recorded to be between 3.5 and 5.4 % per year according to the manual muscle testing and quantitative muscle testing criteria, respectively. Disorders of the respiratory system were the most common cause of death. In another study, 136 patients (57 % males; average age, 61 years at disease onset) were included [14]. During their follow-up, 75 % patients had significant walking difficulties and 37 % used a wheelchair (after a median duration of 14 years from the onset). Compared to these studies, a rather low rate of patients with finger flexor weakness or asymmetric weakness was described in our survey. This could be because the weakness is masked by compensatory mechanisms by lumbricals/FDS or not noticed by physicians without examining the FDP specifically. The cross-sectional nature of our study might affect the lower rate of symptoms. The overall demographic details of patients are similar between Japan and Western countries.

The other limitation of this study was that we should have included in the questionnaires for both patients and caregivers detailed questions on the functional ability to swallow [16] and the form of the consumed food (e.g., whether the meal was minced) in the questionnaire for both the patients and caregivers. Aspiration pneumonia is the common complication for sIBM and can sometimes be fatal. More detailed questions (e.g., how much cheese was eaten per week) with annual follow-up would be helpful in any future study.

## Conclusion

Through our multicenter patient survey and caregiver questionnaire, the phenotypes of sIBM in Japan are similar to those in Western countries at least through a cross-sectional methodology. Many patients described psychological/mental and financial anxiety because of the aged profile of sIBM patients. A follow-up survey is required to reveal the prospective natural history of this disease in Japan.

## Abbreviation

cN-1A: Cytosolic 5′-nucleotidase 1A
FDP: Flexor digitorum profundus
IVIg: Intravenous immunoglobulin
PSL: Prednisolone
sIBM: Sporadic inclusion body myositis
